# Phospholipid scramblases TMEM16F and Xkr8 mediate distinct features of phosphatidylserine (PS) externalization and immune suppression to promote tumor growth

**DOI:** 10.1038/s41420-025-02789-y

**Published:** 2025-11-06

**Authors:** Varsha Gadiyar, Rachael Pulica, Ahmed Aquib, James A. Tranos, Christopher Varsanyi, Trevor Frederick, Ziren Wang, Luis Fernandez Almansa, Lawrence Gaspers, Mariana S. De Lorenzo, Sergei V. Kotenko, Sushil Tripathi, Roger W. Howell, Alok Choudhary, David C. Calianese, Raymond B. Birge

**Affiliations:** 1 Department of Microbiology, Biochemistry and Molecular Genetics, Center for Cell Signaling, Rutgers New Jersey Medical School, Newark, NJ USA; 2 Department of Pharmacology, Physiology, and Neuroscience, Rutgers New Jersey Medical School, Newark, NJ USA; 3 Department of Cell Biology, Rutgers New Jersey Medical School, Newark, NJ USA; 4 Division of Radiation Research, Department of Radiology, Center for Cell Signaling, Rutgers New Jersey Medical School, Newark, NJ USA; 5 International Center for Public Health, Rutgers Health, Newark, NJ USA; 6 Department of Biological Sciences, Seton Hall University, South Orange, NJ USA

**Keywords:** Cancer microenvironment, Phospholipids, Apoptosis

## Abstract

The phospholipid scramblases Xkr8 and TMEM16F externalize phosphatidylserine (PS) by distinct mechanisms. Xkr8 is activated by caspase-mediated proteolytic cleavage and, in synergy with the inactivation of P4-ATPase flippases, results in the irreversible externalization of PS on apoptotic cells and an “eat-me” signal for efferocytosis. In contrast, TMEM16F is a calcium-activated scramblase that reversibly externalizes PS on viable cells via the transient increase in intracellular calcium in live cells. The tumor microenvironment (TME) is abundant with exposed PS, resulting from prolonged oncogenic and metabolic stresses and high apoptotic indexes of tumors. Such chronic PS externalization in the TME has been linked to host immune evasion from interactions of PS with inhibitory PS receptors, such as TAM and TIM family receptors. Here, in an effort to better understand the contributions of apoptotic vs live cell PS-externalization to tumorigenesis and immune evasion, we employed an EO771 orthotopic breast cancer model and genetically ablated Xkr8 and TMEM16F using CRISPR/Cas9. While neither the knockout of Xkr8 nor TMEM16F showed defects in cell intrinsic properties related to proliferation, tumor-sphere formation, and growth factor signaling, both knockouts suppressed tumorigenicity in immune-competent mice, but not in NOD/SCID or RAG-knockout immune-deficient strains. Mechanistically, Xkr8-KO tumors suppressed macrophage-mediated efferocytosis, and TMEM16F-KO suppressed ER stress/calcium-induced PS externalization. Our data support an emerging idea in immune-oncology that constitutive PS externalization, mediated by scramblase dysregulation on tumor cells, supports immune evasion in the tumor microenvironment. This links apoptosis/efferocytosis and oncogenic stress involving calcium dysregulation, contributing to PS-mediated immune escape and cancer progression.

## Introduction

The composition of lipid modalities and their distribution in the plasma membrane lipid bilayer are critical for maintaining the biochemical and biophysical properties of the membrane [1, 2]. In eukaryotic cells, plasma membrane phospholipids are asymmetrically distributed across the membrane, whereby phosphatidylcholine (PC) and sphingomyelin (SM) are mostly confined to the outer leaflet while phosphatidylethanolamine (PE), phosphatidylinositol (PI) and phosphatidylserine (PS) are mostly restricted to the inner leaflet [3]. In resting cells at homeostasis, the negative anionic charge of PS is also important for the recruitment of cytosolic signaling and anchoring proteins with polybasic regions, as well as regulating phospholipid exchange reactions between the plasma membrane and the endoplasmic reticulum via lipid transfer proteins [4–8]. The maintenance of PS asymmetry in healthy cells is primarily regulated by P4-ATPase and related flippases that actively facilitate the vectorial transfer of PS from the outside to the inner membrane, such that, under homeostasis, the PS is almost completely restricted to the intracellular leaflet [9–11].

However, cells can rapidly disrupt PS and phospholipid asymmetry under a variety of physiological conditions and expose PS and PE to the outer cell surface by lipid scramblases. Emblematically, during apoptosis, caspase-mediated activation of the lipid scramblase Xkr8 (and concomitant inactivation of P4 ATPase flippases) results in irreversible PS externalization in the dying cells and signal for the apoptotic cells to the internalized and degraded by efferocytosis [12–14]. By contrast, during cell activation or the transient rise in intracellular calcium, cells can activate the calcium-dependent TMEM16 family of lipid scramblases [15, 16] to transiently and reversibly externalize PS. In both cases, by redirecting PS from the inside of the membrane to the outer membrane, these lipid scramblases effectively switch PS binding of intracellular proteins (i.e., the intracellular PS proteome) to binding extracellular proteins (i.e., the extracellular PS proteome). On PS-positive apoptotic cells, externalized PS interacts with a host of inhibitory PS receptors, including TIMs, TAMs, CD36, and BAI1 that promote efferocytosis [17–20] and tolerance [21–25]. On activated cells, such as T cells, NK cells, dendritic cells (DCs), and macrophages, externalized PS can interact with immune-resolving factors that dampen inflammation to prevent collateral tissue damage [26–28]. Moreover, on platelets and endothelial cells, externalized PS can recruit coagulation factors that facilitate fibrin formation and wound repair [29]. Such PS-interacting proteins convey many physiological and homeostatic regulatory functions that maintain tissue function.

In contrast to the physiological and homeostatic events associated with PS externalization under conditions described above, PS is constitutively externalized in the tumor microenvironment by mechanisms that are still not completely understood [18, 19]. Many highly proliferative tumors have high apoptotic indexes that necessitate excessive efferocytosis, which have been correlated as immune-suppressed tumors with poor survival prognosis and outcomes [30, 31]. In addition, most solid tumors are associated with metabolically stressed and hypoxic vasculature, which externalize PS, leading to the development of PS-targeting monoclonal antibodies (mAbs) such as Bavituximab [32–36]. PS externalization in the tumor microenvironment (TME) has also been associated with exhausted T cells, Tregs, and myeloid suppressor cells [34, 35, 37], as well as the polarization of macrophages towards wound healing M2 phenotypes, as well as maintaining immature DCs [38]. Although many solid tumors display constitutive PS externalization that can be assessed by homing of PS-targeting antibodies [39–41] and Annexin V [42, 43], the mechanisms of PS externalization in the tumor microenvironment are surprisingly not well understood, nor is the composition of PS-positive cells in the TME well defined.

Here, we investigated the contributions of two phospholipid scramblases, Xkr8 and TMEM16F, that are implicated in PS externalization in the context of immune regulation. We hypothesize that tumor cells are one of the major sources of externalized PS in the TME. Using an EO771 luminal B orthotopic breast cancer model, we show that EO771 cells preferentially express both Xkr8 and TMEM16F over other isoforms, and that EO771 tumor-bearing transplanted mice externalized PS in the tumor microenvironments as evident by the homing of PS-targeting mAbs. Subsequently, using CRISPR/Cas9 gene editing to individually knockout Xkr8 or TMEM16F, we show that both PS scramblases directly contribute to immune regulation and tumor growth in immune-competent mice but not in NOD/SCID or Rag1 KO immune-deficient mice. Our data support complex and dual mechanisms for PS externalization in the tumor microenvironment, whereby apoptosis/efferocytosis of growing tumors and calcium-stressed viable tumor cells both likely contribute to PS-mediated immune escape and tumor progression.

## Results

### PS is chronically externalized in the tumor microenvironment in EO771 orthotopically grafted tumor-bearing mice

Previous studies employing an EO771 orthotopic syngeneic breast cancer model showed therapeutic efficacy when either PS-targeting antibodies (Bavituximab) or PS receptors (Mertk) were administered in combination with anti-PD1 checkpoint therapeutics, suggesting an inhibitory PS and PS-Receptor axis that cross-talks to T cells is functional in this model [38, 44, 45]. However, despite the importance of inhibitory PS signaling in the tumor microenvironment, it is still unclear (1) the mechanisms by which PS becomes chronically dysregulated in solid cancers and (2) the cell type(s) that contribute to PS externalization and inhibitory signaling to achieve immune evasion. Here, in an attempt to investigate the most proximal cell-intrinsic events that contribute functionally to PS externalization and immune signaling, we targeted two PS scramblases expressed on the EO771 cells that include the caspase-activated scramblase Xkr8 [13–15] and the calcium-activated scramblase TMEM16F [16, 46, 47]. To verify that the EO771 (orthotopic tumor) model displayed constitutively exposed PS, we first cloned, expressed, and purified PS-targeting antibodies 11.31, 1N11, and Bavituximab from Expi293 cells [48]. We also employed a truncated (GLA + EGF)-Fc (mouse IgG2a) PS-binding Fc fusion domains that preferentially bind to PS (Fig. S1A) [49]. As previously noted, the purified antibodies 1N11 and Bavituximab bind to PS indirectly through β2GPI, whereas 11.31 and Gla-EGF [4] bind directly to PS [48].

To characterize in vivo PS externalization in the tumor microenvironment, we labeled the aforementioned PS targeting antibodies with an NIR dye or radioisotope Zr-89 to track their biodistribution in tumor-bearing mice. In vivo imaging methods, including IVIS and PET/CT, were employed to monitor PS antibody localization in the EO771 tumor-bearing mice (Fig. S1B). IVIS imaging showed in vivo localization of PS targeting antibodies and the Gla-EGF fusion protein to the tumors at 24 (Fig. S1C) and 48 h (Fig. S1D) after intraperitoneal injection, while isotype antibody showed minimal tumor localization. Tumors, liver, spleen lungs, heart and kidneys were harvested at 72 h after injection and subjected to IVIS. Tissue distribution of Bavituximab was seen to be most prominent in tumors and liver, followed by kidney, and least in lungs, spleen and heart (Fig. S1E). Antibody accumulation in the liver and kidneys could be due to immune complex formation. Tumors notably showed high levels of antibody localization of Bavituximab, suggesting that tumors externalize PS, and that the PS is constitutive and available for PS targeting by therapeutic modalities (Fig. S1F).

To verify the above findings, in vivo imaging studies were also conducted with ^89^Zr-labeled direct PS binding antibody 11.31 and assessed using PET/CT imaging. 11.31 and isotype were labeled with ^89^Zr (chosen due to its long half-life of 78.4 h, allowing us to track antibody localization for longer times up to 72 h). PET/CT imaging showed localization of 11.31 to EO771 tumor tissues at 24, 48 and 72 h after injection, compared to isotype (Fig. S1G). As noted, accumulation in the liver was observed in both isotype and 11.31; however, the tumors (circled) showed a notable increased localization of the 11.31 antibody that was sustained for 72 h after injection compared to the isotype (Fig. S1H). Taken together, our results indicate that EO771 tumors harbor robust PS externalization in orthotopic tumor-bearing mice, as previously shown by other groups as well [38].

While PS-targeting antibodies, shown here and elsewhere, are known to localize to the TME in solid cancers, it is still not clear the functional contribution of tumor cells versus tumor vasculature and stromal cells towards immune evasion [18, 50]. Indeed, while previous studies reported that PS antibodies can bind to the tumor vasculature [32, 40, 51], the contribution of tumor cells functioning as a cell-intrinsic event to PS externalization and immune escape mechanisms in the TME is not defined. To investigate the tumor cell-intrinsic role of PS on tumor cells, we explored targeting PS scramblases TMEM16F and Xkr8 specifically on EO771 tumor cells by CRISPR/Cas9 gene editing.

### Ablation of Xkr8 on EO771 tumor cells blocks PS externalization on dying cells without altering cell-intrinsic oncogenic properties

**Fig. 1:**
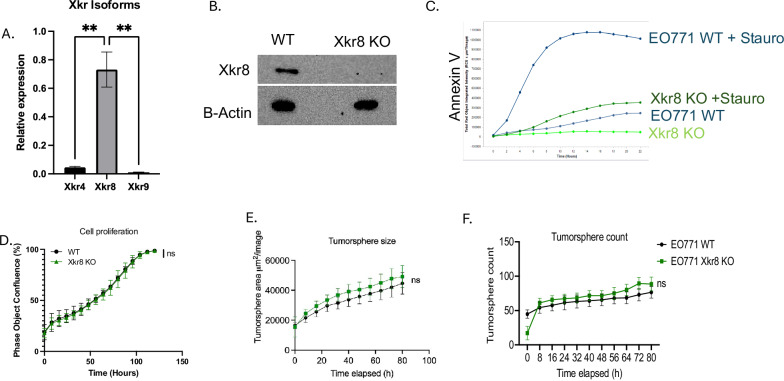
Ablation of Xkr8 on EO771 tumor cells blocks PS externalization during apoptosis without affecting intrinsic oncogenic properties. **A** q-RT-PCR analysis of Xkr isoform expression (Xkr4, Xkr8, Xkr9) in EO771 tumor cells. Xkr8 is the most highly expressed isoform among the Xkr family (Ordinary One-way ANOVA, *n* = 4, *p < 0.05, **p < 0.01, ***p < 0.001, ****p < 0.0001. **B** Validation of Xkr8 knockout (KO) in EO771 tumor cells using western blotting with a custom polyclonal anti-Xkr8 antibody (Abclonal). Complete ablation of Xkr8 is observed in KO clones. **C** Real-time measurement of PS externalization in Xkr8 KO and wild-type (WT) EO771 cells upon apoptosis induction with 100 nM Staurosporine using fluorescently labeled Annexin-V, as measured by Incucyte. **D** Cell proliferation assay comparing Xkr8 KO and WT EO771 tumor cells as measured by bright field phase object confluence (%) by Incucyte. Tumor-sphere formation assay in Matrigel comparing Xkr8 KO and WT EO771 tumor cells was also measured by Incucyte. **E** Quantification of tumor-sphere area μM^2^/Image and **F** tumor sphere counts were compared for Xkr8 KO and WT EO771 tumor-spheres.

To first investigate the expression patterns of Xkr family members including Xkr4, Xkr8, and Xkr9, (all of which contain caspase recognition sites for activation), we screened the EO771 cells by q-RT-PCR (Fig. 1A), Notably, of these isoforms the Xkr8 isoform was predominantly expressed at the mRNA level (Fig. 1A), suggesting a predominant role of Xkr8 in PS scrambling and externalization. To assess biological function, Xkr8 was targeted by CRISPR/Cas9 gene editing, after which single cell clones were selected and passaged. Successful knockout was verified using a custom polyclonal rabbit anti-mouse Xkr8 antibody and by a surveyor PCR assay to interrogate the gene locus (Figs. 1B, S3A). To assess functional impairment of PS externalization during apoptosis, we treated the EO771 Xkr8 KO cells with 100 nM Staurosporine, an ATP kinase inhibitor and inducer of apoptosis, and assessed PS externalization in real time using Incucyte and fluorescently labeled Annexin-V as a function of time. As shown in Fig. 1C, Xkr8 knockout cells showed a dramatic reduction in the intensity of fluorescently labeled Annexin V compared to WT cells, indicating PS externalization can be uncoupled from caspase-mediated apoptosis in the EO771 cells. Interestingly, however, EO771 Xkr8 knockout cells did not show apparent defects in tumor cell proliferation (Fig. 1D) or tumor-sphere formation in Matrigel, as shown by tumor sphere size (Fig. 1E) and tumor sphere count (Fig. 1F), or defects in the immediate Gas6-mediated activation of p-Akt through Axl (Fig. S3B), suggesting that Xkr8 does not impinge on the cell-intrinsic oncogenic features of these cells in cell culture.

### Xkr8 knockout on tumor cells reduces tumor growth in vivo in the orthotopic EO771 breast cancer model

**Fig. 2:**
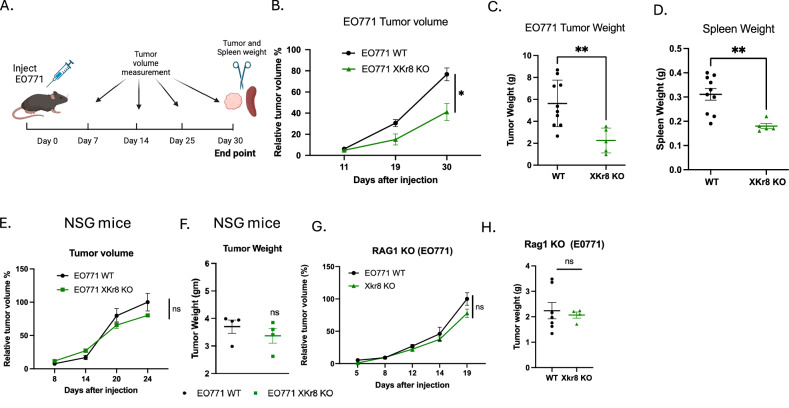
Xkr8 knockout reduces tumor growth in an orthotopic model of breast cancer. **A** Experimental timeline for orthotopic injection of EO771 wild-type (WT) or Xkr8 knockout (KO) tumor cells into the mammary fat pads of C57BL/6 wild-type (WT) mice. Tumor volumes were measured periodically to assess growth. Tumor weights and spleen weights were measured at endpoint. **B** Relative tumor volumes of EO771 WT and Xkr8 KO tumors at various time points (Mean values ± SD, Two-way ANOVA, Mixed effects model, *n* = 15). **C** Tumor weights of EO771 WT and Xkr8 KO tumors at the time of sacrifice point (Mean values ± SD, One-way ANOVA, *n* = 15). **D** Spleen weights of EO771 WT and Xkr8 KO tumor-bearing mice point (Mean values ± SD, One-way ANOVA, *n* = 15). **E** Relative tumor volumes in immune-deficient NSG mice, injected with EO771 WT or Xkr8 KO cells (Mean values ± SD, Two-way ANOVA, Mixed effects model, *n* = 9). **F** Tumor weights in NSG mice injected with EO771 WT or Xkr8 KO cells (Mean values ± SD, One-way ANOVA, *n* = 9). **G** Relative tumor volumes (Mean values ± SD, Two-way ANOVA, Mixed effects model, *n* = 10) and **H** tumor weights (Mean values ± SD, One-way ANOVA, *n* = 9) in Rag1 knockout (KO) mice, which lack mature T and B cells. (*p < 0.05, **p < 0.01, ***p < 0.001, ****p < 0.0001).

To investigate in vivo tumor growth in an immunocompetent mouse model, we used the syngeneic orthotopic breast cancer as previously described [44, 45]. When EO771 WT cells or EO771 Xkr8 KO cells were injected into mammary gland fat pads of C57BL/6 WT mice for longitudinal tumor volume studies (Fig. 2A), the Xkr8 KO tumors exhibited significant reduction in tumor growth compared to WT tumors, as evident by relative tumor volume (Fig. 2B), tumor weight (Fig. 2C) and spleen weight (Fig. 2D). To examine the dependence of an immune component in the tumor immune microenvironment, we compared the immunocompetent background above (Fig. 2A–D) with NSG and Rag1 KO immune-deficient mouse models. When WT EO771 or Xkr8 KO cells were injected into NSG mice, which have a dysfunctional adaptive immune system, the KO tumors grew at a similar rate compared to the EO771 WT tumors, as shown by relative tumor volume, compared to WT at endpoint (Fig. 2E) and tumor weight (Fig. 2F). Similar observations were noted in the Rag1 KO mice, which do not have matured T and B cells, whereby the Xkr8 KO tumors grew similarly as WT evident by both tumor volume (Fig. 2G) and tumor weight (Fig. 2H). Together, these observations suggest an active role of an immune component in the tumor regression in Xkr8 KO tumors.

### Ablation of TMEM16F on EO771 tumor cells blocks PS externalization on calcium-stressed cells without altering cell-intrinsic oncogenic properties in EO771 cells

**Fig. 3:**
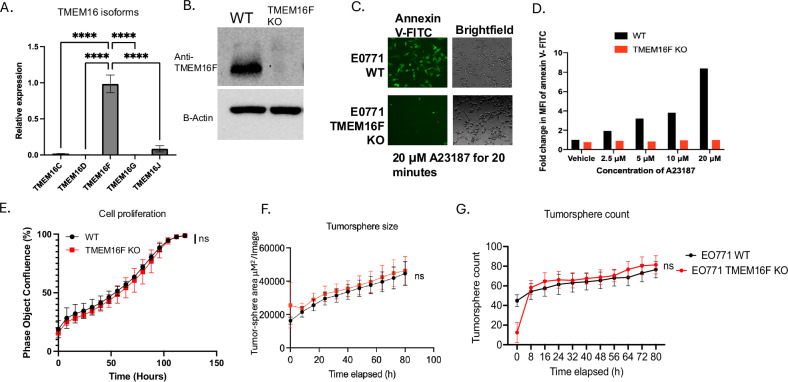
TMEM16F knockout prevents PS externalization in response to calcium stress without altering intrinsic oncogenic properties. **A** q-RT-PCR analysis showing the relative expression of TMEM16 isoforms (16C, 16D, 16F, 16G, and 16J) in EO771 cells (Mean Relative gene expression values ± SD, Ordinary One-way ANOVA, *n* = 3, *p < 0.05, **p < 0.01, ***p < 0.001, ****p < 0.0001). **B** Western blot validation of TMEM16F knockout (KO) in EO771 cells. The absence of TMEM16F is confirmed in the KO clones using a custom antibody (courtesy of Lily Jan, UCSF). **C** Fluorescent microscopic analysis of PS externalization in response to calcium influx using A23187 (calcium ionophore) and Annexin V-FITC staining. EO771 wild-type (WT) cells show robust PS externalization, while TMEM16F KO cells exhibit significantly reduced PS externalization. **D** Fold change in MFI for Annexin V-PE staining by flow cytometry of PS externalization in EO771 WT and TMEM16F KO cells upon treatment with A23187. **E** Cell proliferation assay comparing EO771 WT and TMEM16F KO cells using Incucyte, as measured by bright field phase object confluence % by Incucyte. Tumor-sphere formation assay in Matrigel comparing TMEM16F KO and WT EO771 tumor cells was also measured by Incucyte. **F** Quantification of tumor-sphere area μM^2^/Image and **G** tumor sphere counts were compared for TMEM16F KO and WT EO771 tumor-spheres.

Having established Xkr8 knockout cells in vitro and in vivo EO771 model, we next explored the effects of a calcium-activated scramblase in a comparison to Xkr8. Among the TMEM16 isoforms (16C, 16D, 16F, 16G and 16J), TMEM16F had robust expression at the mRNA level in EO771 cells (Fig. 3A) and therefore was assessed for functional outcomes. Towards this goal, and analogous to the Xkr8 strategy, we depleted TMEM16F in EO771 cells using CRISPR-Cas9 gene editing and sorted single cell clones. Validation of the TMEM16F KOs was assessed using Western blot with an antibody specific for TMEM16F (Antibody courtesy Lily Jan, UCSF) (Fig. 3B). To examine the effects of TMEM16F on calcium-mediated PS externalization. WT and TMEM16F knockout EO771 cells were treated with the calcium ionophore A23187, after which PS externalization in response to calcium influx was monitored using Annexin V-FITC binding by microscopy (Fig. 3C) or flow cytometry (Fig. 3D). Xkr8 KO cells retained their ability to externalize PS in response to calcium ionophore treatment (Fig. S3H). Notably, while the EO771 WT cells externalized PS within 20 minutes, by contrast, the TMEM16F KO cells showed a dramatic reduction and marginalized PS externalization under these conditions (Fig. 3C, D). Analogous to the assessment of Xkr8 in cell-intrinsic oncogenic features, TMEM16F knockout cells also did not show defects in tumor cell proliferation (Fig. 3E) or tumor-sphere formation in Matrigel, as shown by tumor-sphere size (Fig. 3F) and tumor-sphere count (Fig. 3G), or defects in the immediate Gas6-mediated activation of Akt (Fig. S3B).

### TMEM16F KO on tumor cells reduces tumor growth in the EO771 orthotopic model of breast cancer

**Fig. 4:**
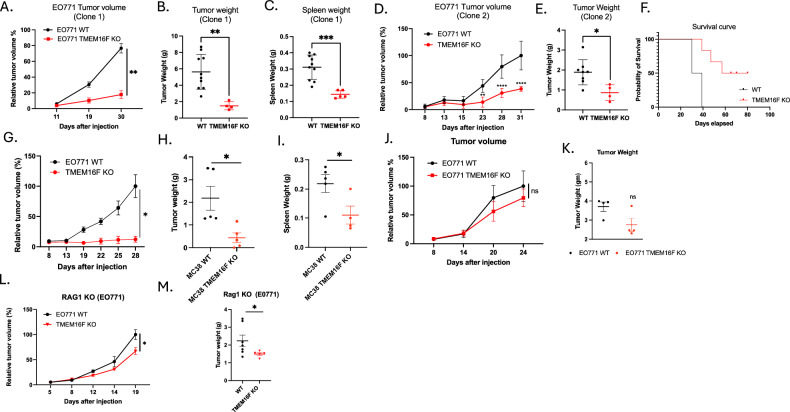
TMEM16F knockout reduces tumor growth in syngeneic orthotopic models of breast and colon cancer. **A** Relative tumor volume % from C57BL/6 WT mice injected with EO771 WT or TMEM16F KO cells into mammary fat pads (Mean values ± SD, Two-way ANOVA, Mixed effects model, *n* = 15). **B** Tumor weight (g) measurements from the same C57BL/6 WT mice showing reduced tumor weight in TMEM16F KO tumors compared to WT tumors (Mean values ± SD, One-way ANOVA, *n* = 15). **C** Spleen weight (g) measurements from C57BL/6 WT mice injected with EO771 WT or TMEM16F KO cells (Mean values ± SD, One-way ANOVA, *n* = 15). **D** Relative tumor volume % (Mean values ± SD, Two-way ANOVA, Mixed effects model, *n* = 10), **E** tumor weight (g) (Mean values ± SD, One-way ANOVA, *n* = 10) and **F** spleen weight (g) (Mean values ± SD, One-way ANOVA) measurements from an independent clone: 2 of EO771 TMEM16F KO cells, showing consistent reduction in tumor growth compared to WT tumors. **F** Kaplan–Meier survival curve showing improved survival in mice bearing EO771 TMEM16F KO tumors compared to those with WT tumors (*n* = 10). **G** Relative tumor volume (Mean values ± SD, Two-way ANOVA, Mixed effects model, *n* = 10). **H** tumor weight (Mean values ± SD, One-way ANOVA, *n* = 10), and **I** spleen weight measurements from MC38 colon cancer model in C57BL/6 WT mice (Mean values ± SD, One-way ANOVA, *n* = 10). **J** Relative tumor volume (Mean values ± SD, Two-way ANOVA, *n* = 9) and **K** tumor weight (g) (Mean values ± SD, One-way ANOVA, *n* = 9) measurements in NSG mice injected with EO771 WT or TMEM16F KO cells, showing no significant difference between the two groups. **L** Relative tumor volume (Mean values ± SD, Two-way ANOVA, Mixed effects model, *n* = 10)and **M** tumor weight measurements in Rag1 KO mice injected with EO771 WT or TMEM16F KO cells (Mean values ± SD, One-way ANOVA, *n* = 15) (*p < 0.05, **p < 0.01, ***p < 0.001, ****p < 0.0001).

To assess the effects of TMEM16F knockout on tumor growth and the tumor microenvironment and compare effects to those of the Xkr8 knockout model described above (Fig. 2B–D), EO771 WT cells or EO771 TMEM16F KO cells were transplanted orthotopically into the mammary gland fat pads of C57BL/6 WT mice. Interestingly, in a similar capacity to Xkr8, the TMEM16F KO tumors also showed significant reduction in tumor growth compared to WT tumors (employing 2 independent clones for validation), as evident by the decrease in tumor volume (Fig. 4A, D), tumor weight (Fig. 4B, E) and spleen weight (Fig. 4C), as well as increased overall survival of mice with TMEM16F KO tumors, as indicated by a Kaplan–Meier curve (Fig. 4F). Similar results were obtained using an MC38 flank colon cancer model, whereby TMEM16F knockout also reduced flank tumor growth, as observed by tumor volume (Fig. 4G), tumor weight (Fig. 4H) and spleen weight (Fig. 4I). These data demonstrate that TMEM16F affects tumor progression in both orthotopic and flank models. However, TMEM16F KO and WT EO771 tumor growth were comparable in immunocompromised NSG mice, as evident by no significant reduction in the tumor volume (Fig. 4J) and tumor weight (Fig. 4K). Interestingly, unlike the Xkr8 knockout phenotype, the TMEM16F KO tumors showed a slight reduction in tumor growth in Rag1 KO mice (which lack mature T and B lymphocytes), with modest effects in tumor volume (Fig. 4L) and tumor weight (Fig. 4M), possibly suggesting that tumor reduction due to Xkr8 KO is more dependent on the mature T and B cells, whereas, in the TMEM16F KO tumors, there is a combined effect of the immune system and other factors, possibly, angiogenesis and modulation of the extracellular matrix, driving the reduction in tumor progression.

### Distinct phenotypic outcomes in the Xkr8 and TMEM16F knockout EO771 cells

The pioneering studies by Nagata and colleagues described the distinct biochemical and molecular features of Xkr8 and TMEM16F as caspase and calcium-activated scramblases, respectively. This interpretation is supported in the EO771 model employed here, showing that the EO771 cells lacking Xkr8 showed a reduction in externalizing PS during *Staurosporine* treatment (caspase-mediated apoptosis), while the EO771 cells lacking TMEM16F expression showed defective PS externalization during ionophore (calcium)-induced cytosolic calcium elevation.

**Fig. 5:**
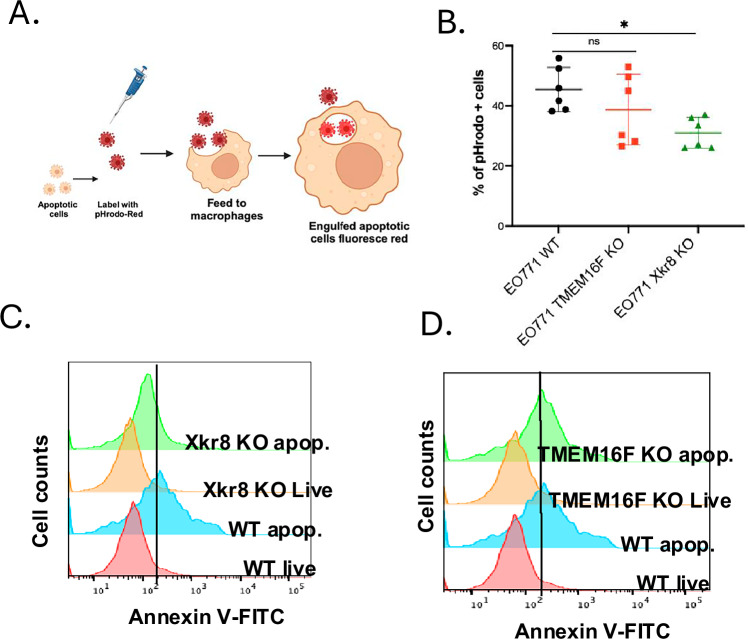
Apoptotic tumor cells induce efferocytosis by Xkr8. **A** Schematic showing the workflow for efferocytosis assays, wherein apoptotic EO771 cells (WT, TMEM16F KO and Xkr8 KO) were labeled with pHrodo red, fed to bone marrow-derived macrophages, and after 3 hours, % of uptake was measured by flow cytometry. **B** Rate of efferocytosis as shown by % of pHrodo red + macrophage cells (Mean ± SD, One-way ANOVA, *n* = 6 *p < 0.05, **p < 0.01, ***p < 0.001, ****p < 0.0001). **C** PS externalization was disrupted in Xkr8 KO apoptotic cells, but not in WT and TMEM16F KO apoptotic cells (**D**), providing evidence that Xkr8-mediated PS externalization is essential for efficient recognition and phagocytosis by BMDMs.

To access the mechanistic and functional consequences of the scramblase knockouts, we examined in vitro efferocytosis using pHrodo-labeled apoptotic EO771 WT, Xkr8 KO and TMEM16F KO cells when co-cultured with bone marrow-derived macrophages (BMDMs) (Fig. 5A). As indicated in Fig. 5B, whereas the BMDMs efficiently engulfed apoptotic WT and apoptotic TMEM16F KO cells, efferocytosis was notedly reduced when Xkr8 KO apoptotic cells were co-cultured with macrophages. TMEM16F KO apoptotic cells were efficiently engulfed by BMDMs, suggesting distinct functions for scramblases, showing that Xkr8 mediated PS externalization on apoptotic tumor cells is essential for recognition by BMDMs (Fig. 5B). Moreover, and analogous to the observations in Fig. 1C, when assessed by flow cytometry for Annexin V-FITC, only the Xkr8 KO cells, but not the TMEM16F KO cells, showed suppression in apoptosis-mediated PS externalization (Fig. 5C, D).

### Intracellular calcium regulation and PS externalization in tumor cells

**Fig. 6:**
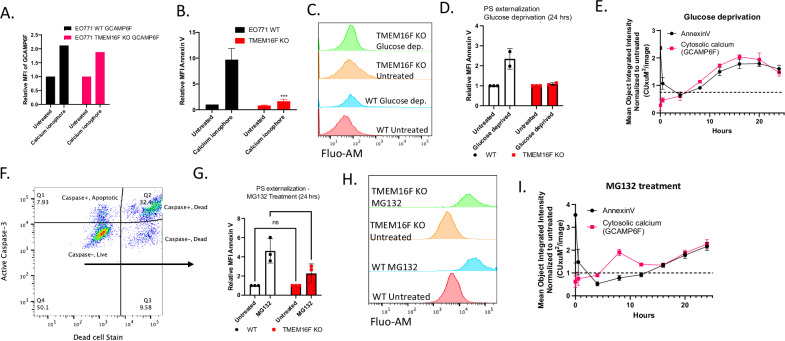
Intracellular calcium regulation and PS externalization in tumor cells. **A** Intracellular calcium levels were upregulated in both WT and TMEM16F KO EO771 cells upon treatment with calcium ionophore, as indicated by the increased calmodulin-GFP fluorescence. **B** PS externalization as measured by relative MFI of Annexin V staining in calcium ionophore-treated WT and TMEM16F KO cells (Mean values ± SD, One-way ANOVA, *n* = 3 *p < 0.05, **p < 0.01, ***p < 0.001, ****p < 0.0001). **C** Flow plots showing intracellular calcium levels as shown by Fluo-AM staining. **D** PS externalization as measured by relative MFI of Annexin V staining in WT and TMEM16F KO cells exposed to glucose deprivation for 24 h (*n* = 2). **E** Live cell imaging with Incucyte shows calcium upregulation and PS externalization (Annexin V) for up to 24 h with glucose deprivation. **F** Gating strategy shows that PS externalization was measured on the live cells that stained negative for active caspase 3, as well as the viability stain. **G** PS externalization on cells treated with low dose MG132, a proteasome inhibitor, as shown by relative MFI of annexin V staining (Mean values ± SD, One-way ANOVA, *n* = 3 *p < 0.05, **p < 0.01, ***p < 0.001, ****p < 0.0001). **H** Flow plots showing intracellular calcium levels upon MG132 staining as shown by Fluo-AM staining. **I** Live cell imaging with Incucyte showed calcium upregulation and PS externalization (Annexin V) for up to 24 hours MG132 treatment.

The findings that TMEM16F KO still retained their capacity to undergo caspase-mediated cell death and PS externalization suggest TMEM16F functions at a distinct level that impinges on calcium-mediated PS externalization. To interrogate the relationship between elevated intracellular calcium and calcium-mediated PS externalization, we generated a calcium reporter cell line by stably transfecting WT or TMEM16F knockout EO771 cells with a plasmid expressing a genetically encoded calcium indicator, GCaMP6f, targeted to the cytosol (Fig. S3J). The GCaMP family of calcium indicators was developed from circularly permutated green fluorescent protein (cpGFP) linked to an engineered calmodulin (CaM) and a CaM-binding M13 peptide. The binding of calcium to calmodulin (Kd ≈ 380 nm) triggers a conformational change within the cpGFP chromophore, resulting in a large increase in fluorescence intensity at 485-nm excitation [52]. As noted in Fig. 6A, while intracellular calcium is equally upregulated in the WT and TMEM16F KO cells upon treatment with calcium ionophore, the PS externalization was severely impaired in the TMEM16F KO cells (Fig. 6B), but not the WT cells, indicating that PS can be functionally dissociated from the elevated intracellular calcium by ablating TMEM16F.

Subsequently, to phenocopy effects of oncogenic endoplasmic (ER) stress, a known inducer of dysregulated and elevated cytosolic calcium, we employed two activators of ER stress that included the treatment of cells with a low dose of proteasomal inhibitor MG132, or the exposure to cells to conditions of glucose deprivation, that result in altered protein folding and protein glycosylation in the ER, respectively [53–58]. As shown in panels Fig. 6C–E (glucose deprivation), these conditions led to increased cellular calcium and PS externalization on live caspase negative, 7AAD negative gated cells (Fig. 6F). Similarly, treatment with low dose MG132, a proteasome inhibitor increased intracellular calcium levels in live cells (Fig. 6G–I), also induced PS externalization via TMEM16F (Fig. 6G–I). Live cell imaging with Incucyte reveals that glucose deprivation and MG132 treatments induce sustained cytosolic calcium increases and PS externalization for up to 24 h (Fig. 6H, I). Notably, in panels 6E and 6I, a protracted time course (10–20 hours) showed calcium elevation and PS externalization were phenocopied (see Fig. S3D, E). Moreover, when these cells were orthotopically injected into mice, elevated calcium was observed associated with the tumor mass (Fig. S3G). Taken together, these studies suggest that knockout of TMEM16F suppressed ER stress-mediated calcium dysregulation in conditions aimed to mimic oncogenic stress.

### Effects of scramblase KO on the tumor microenvironment

**Fig. 7:**
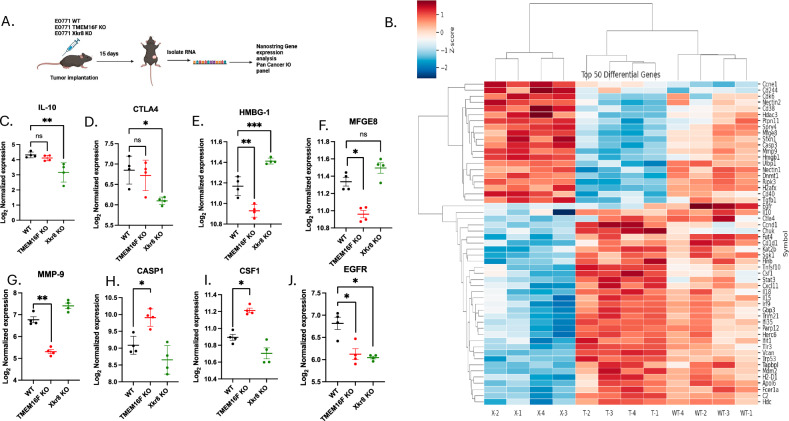
TMEM16F and XKr8 KO affect the TME through distinct pathways. **A** Schematic of the Nanostring Gene Expression Analysis used to assess the tumor immune microenvironment (TME) in tumors grown in C57BL6 WT mice at day 15 after injection. **B** Clustered heat map showing the top 50 differentially regulated genes in Xkr8 KO and TMEM16F KO tumors compared to EO771 WT tumors is shown (*n* = 12, genes for *p*-values < 0.05 are shown and were calculated using ANOVA). WT-1,2,3,4, T-1,2,3,4 and X-1,2,3,4 correspond to EO771 WT, TMEM16F KO and Xkr8 KO tumors, respectively. Gene expression analysis showed downregulation of IL-10 (**C**), a cytokine released after efferocytosis, and CTLA-4 (**D**), an immune checkpoint involved in immune suppression in Xkr8 KO tumors. In contrast, HMGB1, a damage-associated molecular pattern (DAMP) released during immunogenic cell death, was upregulated in Xkr8 KO tumors (**E**). **F**–**I** TMEM16F KO tumors exhibited downregulation of MFGE8 (**F**), a protein involved in PS binding and efferocytosis, and MMP9 (**G**), which is involved in matrix remodeling and cancer progression. Caspase-1 (**H**) and CSF-1 (**I**) were upregulated in TMEM16F KO tumors, indicating increased cell death and macrophage infiltration. **J** Both Xkr8 KO and TMEM16F KO tumors showed downregulation of EGFR expression, suggesting an impact on EGFR-expressing cell populations (Mean log_2_ normalized expression values ± SD, Two-way ANOVA, Mixed effects model, *n* = 12 *p < 0.05, **p < 0.01, ***p < 0.001, ****p < 0.0001).

The tumor studies in the immune-deficient mice suggested a role for the tumor immune microenvironment in the anti-tumor effect of the scramblase KO tumors. To characterize this further, we used NanoString Gene Expression Analysis to assess differentially regulated genes in the TME. We isolated RNA from tumors grown in C57BL/6 WT mice at day 15 and assessed gene expression using the mouse Pan Cancer IO 360 panel (Fig. 7A). Figure shows differentially expressed genes in Xkr8 KO and TMEM16F KO tumors compared to EO771 WT tumors respectively (Fig. 7B). Pathway analysis determined that certain oncogenic pathways, such as angiogenesis, cell proliferation, matrix remodeling and metastasis, Notch signaling, immune cell adhesion and migration are commonly downregulated in both TMEM16F and Xkr8 KO tumors, compared to WT tumors. Upon dissecting individual gene expression, we observed that TMEM16F KO and Xkr8 KO differentially regulate a unique set of genes. In Xkr8 KO tumors, we observed downregulation of IL-10 (Fig. 7C), a classical cytokine released after efferocytosis. CTLA-4, an immune checkpoint, was also seen to be downregulated in Xkr8 KO tumors, pointing to reduced immune-suppression in these tumors (Fig. 7D). However, HMGB1, a classical DAMP that is secreted when cells undergo immunogenic cell death or secondary necrosis was observed to be upregulated in Xkr8 KO tumors (Fig. 7E). The reduced IL-10 and increased HMGB1 in Xkr8 KO tumors suggest possibly reduced efferocytosis in vivo, consistent with the in vitro findings, and that un-cleared apoptotic cells increase inflammation.

**Fig. 8:**
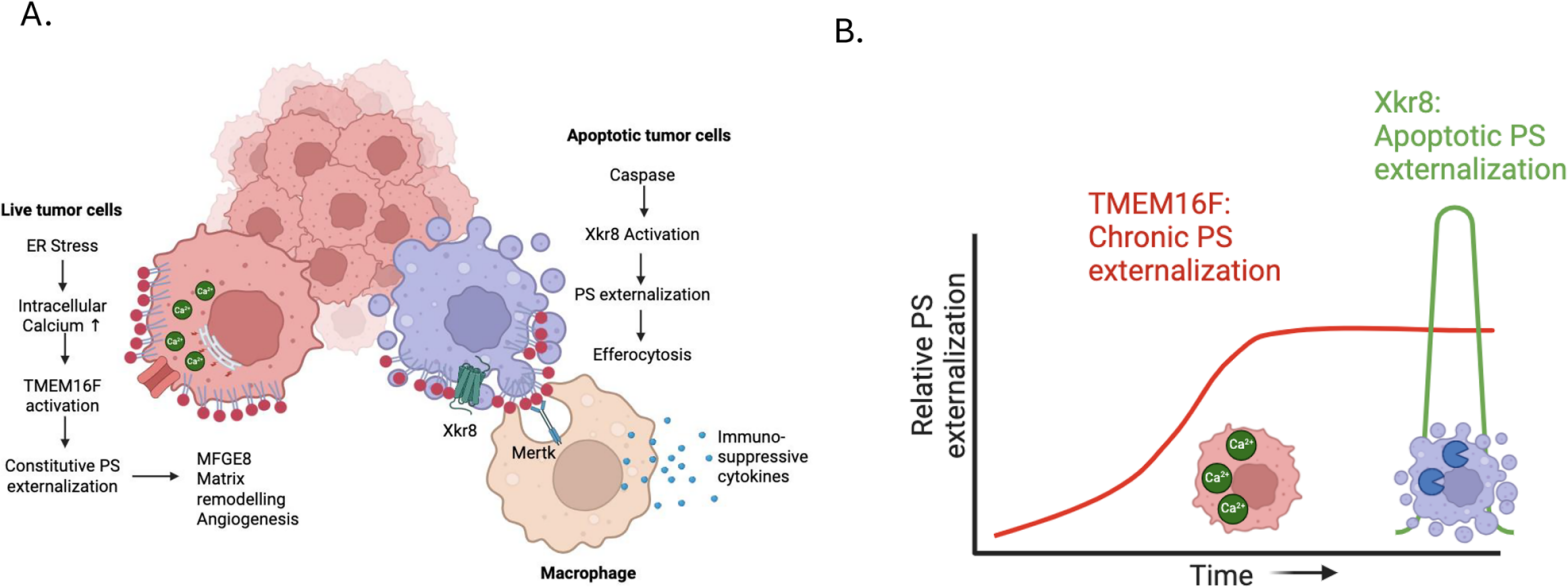
PS externalization on tumor cells by scramblases Xkr8 and TMEM16F mediates pro-tumorigenic effects by distinct pathways. **A** The working model shows that PS externalization by scramblases Xkr8 and TMEM16F mediates pro-tumorigenic effects by unique pathways, wherein Xkr8 upregulates PS exposure on apoptotic cells, leading to increased efferocytosis and secretion of immune-suppressive cytokines, whereas TMEM16F is regulated by calcium stress in live cells, leading to MFGE8-mediated matrix remodeling. **B** Temporal activation of the scramblases can be explained as such: TMEM16F is activated chronically in live cells undergoing chronic ER stress and upregulated intracellular calcium, whereas Xkr8 activation is a late signal induced by caspase-mediated cell death.

Comparing EO771 WT and TMEM16F KO tumors revealed a unique differential gene expression, different from that of Xkr8 KO tumors. TMEM16F KO tumors significantly reduced MFGE8 expression (Fig. 7F), which binds PS binding protein by its C2 domain and is a ligand for α_v_β_3_ and α_v_β_5_ integrins. TMEM16F KO tumors also showed a reduction in MMP9 (Fig. 7G), which exacerbates cancer progression by degrading extracellular matrix proteins. Potentially consistent with this idea, the Xkr8 knockout cells had less metastasis in the NSG mice (Fig. S3C). Caspase-1 and CSF-1 were also upregulated in TMEM16F KO tumors, indicating increased cell death and macrophage infiltration, respectively (Fig. 7H, I). EGFR was the common gene downregulated in both scramblase KOs, indicating that reducing PS externalization was affecting EGFR-expressing cell populations (Fig. 7J). Notably, no differences in immune cell infiltration were noted at this time point, perhaps due to a lack of PS externalization in the EO771 tumors at this time point. Taken together, while both scramblases affected tumor growth in immunocompetent mice, the mechanisms by which Xkr8 and TMEM16F impinge on immune evasion are likely distinct (Fig. 8A).

## Discussion

Dysregulated PS externalization has been documented in a wide array of solid tumor types as evident by both the homing of PS-targeting monoclonal antibodies, beta-bodies, Annexins, and cationic scaffolds/peptoids to the tumor microenvironments [18, 37, 42, 50], as well as functional studies showing therapeutic utility of PS-targeting antibodies [40, 41, 51, 59] and anti-PS-Receptors (Mertk) [44, 60] in immune oncology, often acting in synergy with checkpoint inhibitors such as anti-PD-1. However, despite the promise of targeting PS as a universal modality in the immune-oncology of solid cancers, neither the mechanisms of PS externalization in the complex tumor microenvironment nor the repertoire of cell types that mediate persistent PS externalization are currently well articulated. In the current study, we employed the EO771 syngeneic tumor model that has been previously shown to be responsive to both PS-targeting Abs (Bavituximab) [38] as well as anti-Mertk (a PS receptor belonging to the TAM family) [44]. We show that EO771 tumor-bearing mice with palpable tumors are targeted by two distinct PS-targeting modalities (Bavituximab and 11.31), as well as a Gla-containing PS-targeting protein, indicating the PS constitutively externalized during tumor growth. Furthermore, EO771 cells express the two main PS scramblase isoforms Xkr8 (caspase-activated) and TMEM16F (stress and calcium-activated), permitting molecular dissociation of the cancer cell-intrinsic events, side-by-side, associated with apoptosis/efferocytosis (Xkr8) from the events associated with calcium dysregulation and PS externalization on oncogenic viable cells (TMEM16F). Our results provide evidence that both Xkr8 and TMEM16F knockout tumor cells suppress in vivo tumor growth in immunocompetent mice but not in NOD/SCID or Rag1 KO immuno-incompetent mice. Our data support a complex and dual mechanism for PS externalization in the tumor microenvironment, whereby apoptosis/efferocytosis of growing tumors and calcium-stressed viable tumor cells both likely contribute to PS-mediated immune escape and tumor progression.

Although the biochemical mechanisms by which loss of Xkr8 and TMEM16F impinge on tumor growth are still not clear, our results are consistent with a recent study by Chen and colleagues showing that co-delivery of an Xkr8 small interference RNA (siRNA) with platinum chemotherapy (to enhance apoptosis) showed increased therapeutic efficacy in an orthotopic pancreatic tumor model with an increase of proliferative NK cells and activated macrophages infiltration in the tumor microenvironment [61]. Moreover, using single-cell RNAseq, these authors showed increased infiltration of CD8+ cytotoxic T cells and reduced exhausted T cells, suggesting that the loss of Xkr8 promoted a type of immunogenic death response, perhaps involving the cross-presentation of tumor antigens by APCs. For example, Xkr8-deficient tumor cells showed enhanced secretion of cyclic GAMP to activate STING on neighboring cells. In our studies, the Xkr8 was only deleted on the tumor cells, not other cells in the tumor microenvironment, nor did we use external chemotherapeutics to enhance tumor cell death in the in vivo studies. As such, we propose a model in which a finite number of proliferating tumor cells die by apoptosis in the tumor microenvironment and are actively engulfed by the tumor-associated macrophages and possibly other bystander phagocytes. Indeed, consistent with this idea, the EO771 cells with Xkr8 KO fail to externalize PS in vitro when treated with staurosporine, and these cells display impaired in vitro efferocytosis when co-cultured with bone marrow-derived macrophages. Additionally, the Nanostring gene expression profiling revealed an efferocytic signature including decreased PS opsonin molecule MFG-E8, which has been shown to link apoptotic cells to PS-mediated efferocytosis with integrins [62–64]. Interestingly, since recent studies suggest that chemotherapies can induce Xkr8 at both the transcriptional and translation levels, further studies are needed to assess the synergy of Xkr8 targeting modalities with chemotherapies or inducers of immunogenic death.

While the increased immunogenic outcomes associated with loss of Xkr8 scramblase function can be rationalized by impaired efferocytosis and potential secondary necrosis and activation of DAMPs, the mechanisms by which tumor growth and immune activation in the TMEM16F KO EO771 cells are more elusive but nonetheless interesting. In vitro, the TMEM16F KO cells are impaired in calcium-mediated PS externalization, suggesting that prolonged calcium signaling occurs on the viable tumor cells in the tumor microenvironment. It has long been recognized that dysregulated calcium is associated with oncogenic stress, including mitochondrial and ER stress, as well as hypoxia and glucose deprivation [53]. We posit that one or more of these signals contribute to altered and prolonged calcium signaling in tumor cells, and these cells can engage inhibitory PS receptors on immune cells in the tumor microenvironment. Indeed, very interesting studies by Wang and colleagues recently established constitutively “PS^out^” live tumor cells by knocking out the TMEM30 (CDC50A) subunit of P4 ATPases, a family of flippases that vectorially transfer PS from the outer surface to the inner surface of the plasma membrane [65]. PS^out^ tumors developed more aggressively than wild-type (WT) tumors, showing M2 polarized tumor-associated macrophages (TAMs) and fewer tumor-antigen-specific T cells. These studies, and previous studies by Rothlin and colleagues, showing that externalized PS on live cells can inhibit TIM-3 on DCs to downregulate co-stimulatory receptors support the idea that PS externalization on live cells is an inhibitory immune signal [27, 66]. In the studies here, by limiting the TMEM16F-mediated PS externalization on the EO771 tumor cells, these studies support the idea that tumor cells, as a cell-intrinsic event and mechanism, can mediate in part immune evasion that occurs in the tumor microenvironment.

While our present studies are consistent with the idea of cell-intrinsic mediated events on the dying and viable tumor cells, via the cell-intrinsic loss of either Xkr8 or TMEM16F, it is still unclear what other cell types may contribute to PS externalization in the complex tumor microenvironment. For example, in the original conception of Bavituximab, a PS-targeting mAb, Thorpe and colleagues conceptualized that hypoxia and neo-vascularization promoted PS externalization on vascular endothelial cells in the tumor microenvironment, suggesting that Bavituximab, at least in part, functions as an anti-vascular agent [32, 51, 67]. In the case of hypoxia, the recent studies by Makela and colleagues showed that populations of CD44+ cancer stem cells become PS-positive and can be targeted by novel PS-directed payloads [68]. In the EO771 model here, we did not observe hypoxia-mediated increased PS externalization, but we did not investigate a stem cell pool (Fig. S3F). Further studies are also needed to address the effects of hypoxia and glucose deprivation in the tumor microenvironment on PS externalization on immune cells such as macrophages, cytotoxic T cells, central memory cells, and T regulatory cells that can lead to immune exhaustion, and whether PS externalization can feedback and inhibit DCs to suppress co-stimulatory molecules and antigen presentation, as alluded to above. Clearly, while the tumor-centric approach to knockout scramblases provides proof-of-concept that PS externalization on the tumor cells can drive immune evasion, further studies using CITE-seq to identify the subsets and cell types that contribute to PS in the tumor environment are clearly warranted and meritorious.

Another emerging area of PS biology that deserves further attention is whether tumor cells upregulate or alter the expression of Xkr8 or TMEM16F as a cell-intrinsic or driver event in host immune evasion and tumor progression. For example, surveying the Kaplan–Meier database suggests that expression levels of Xkr8 or TMEM16F are associated with poorer overall survival in breast cancer (Fig. S2A, B). Clearly, identification of signaling pathways that lead to activation and/or upregulation of Xkr8 and TMEM16F might lead to “oncogenic hubs” that link signal transduction to PS-mediated immune escape. As noted above, Xkr8 upregulation has been observed in solid tumors following chemotherapy and radiation therapy, possibly as an adaptation to oncogenic stress [61, 65]. Moreover, phosphorylation at the carboxyl termini region of Xkr8 has been shown to activate the scramblase activity, potentially linking oncogenic kinases with PS externalization [69, 70]. Similarly, oncogenic pathways that activate phospholipase C are long known to increase cellular IP3 and increase cytosolic calcium, potentially linking tyrosine kinase signaling to immune evasion. Finally, in recent years, emerging evidence suggests that activating mutations in flippases and scramblases can constitutively inactivate or activate enzymatic activity, possibly as an oncogenic event that leads to intrinsic host immune evasion [50]. Further studies are needed to assess the frequency of oncogenic mutations that lead to PS externalization on tumor cells in this emerging research field.

While the present study shows proof-of-concept that loss of function of the caspase-activated and calcium-activated scramblases and impinge on host immunity, many questions remain about the physiological context of how these scramblases are typically regulated in the tumor microenvironment. However, these studies support the further development of scramblase-modifying modalities as a new line of therapeutic intervention that may have benefits in immune-oncology.

## Methods

### Cell culture

All cells except Expi293 were cultured in a humidified incubator at 37 °C and 5% CO_2_. EO771 and HEK293T cells were cultured in DMEM ([+] 4.5 g/L glucose, L-glutamine [-] sodium pyruvate) supplemented with 10% FBS and 1% Penicillin/Streptomycin in 100 mm cell culture-treated dishes. MC38 and B16 F-10 cells were cultured in DMEM ([+] 4.5 g/L glucose, L-glutamine [-] sodium pyruvate) supplemented with 10% FBS, 1% Penicillin/Streptomycin and additional 5% L-Glutamine Streptomycin in 100 mm cell culture-treated dishes. Jurkat cells, W3-I1dm and W3-CDC50A^ED29^ cells (suspension cells) were cultured in RPMI supplemented with 10% FBS and 1% Penicillin/Streptomycin in T75 culture flasks. BMDMs (Bone marrow-derived macrophages) were cultured in IMDM supplemented with 10% heat-inactivated FBS, 1% Penicillin/Streptomycin and 20 ng/mL of recombinant mouse M-CSF. Expi293 cells were cultured in Expi293 expression media in Erlenmeyer flasks in a humidified incubator at 37 °C and 8% CO_2_ on a shaker at 110 rpm. Cell treatments were done for 24 hours with 10 μM MG132, or glucose-free DMEM for glucose deprivation.

### BMDM isolation

Bone marrow-derived macrophages were isolated from the tibia and femurs of 8–12-week-old C57BL/6 WT mice. The tibia and femurs were isolated and washed for 15 seconds each, once in 70% ethanol and then in PBS, following which their ends were cut to reveal the bone marrow. The bones were placed cut end facing down in a 0.5 mL Eppendorf tube with a hole pierced with an 18 G needle, which was placed in a 1.5 mL Eppendorf tube containing 80 μL of BMDM media. The tubes were then centrifuged at 15,000×*g* to allow the bone marrow to precipitate into the 1.5 mL Eppendorf tube. The cell pellet was treated with 5 mL of RBC lysis buffer for 5 min at room temperature, later quenching with BMDM media. The cells were collected by centrifuging at 300×*g* for 5 min and strained using a 70-micron cell strainer. The cells were counted using trypan blue exclusion using a hemocytometer and were plated in 150 mm cell culture-treated plates at 10^7^ viable cells per plate, in a total volume of 30 mL. Recombinant murine M-CSF (Biolegend-576406) was added at a final concentration of 20 ng/mL. On day 3 of differentiation, 15 mL of the cell culture medium was removed and replaced with 15 mL of fresh M-CSF-containing medium. BMDMs were fully differentiated on day 7. Differentiated BMDMs were detached gently using a scraper and re-plated into 6-well, 12-well or 24-well plates based on experimental needs.

### Expression constructs

The protein sequences for Bavituximab (US8956616B2), 1N11 (US20180289771A1), and 11.31 (US20110318360A1) were retrieved from their respective patents, and the corresponding DNA sequences were obtained through reverse translation using the Expasy Translate tool. Gene fragments for the VH (variable heavy) and VL (variable light) chains of these antibodies are designed with overlapping regions of approximately 18 bp to ensure compatibility with the respective backbone vectors, and are synthesized by Genewiz (Azenta Life Sciences). The pVITRO1-M80-F2-human IgG1/κ and pVITRO1-dV-humanIgG1/λ plasmids are isolated from 5 mL bacterial cultures using the Qiagen Miniprep kit, with the backbone regions amplified by PCR using Q5 high-fidelity polymerase. The PCR products are analyzed by agarose gel electrophoresis and purified with a PCR purification kit (Qiagen). The NEB Builder HiFi assembly cloning kit is used to ligate the four DNA fragments (backbone regions and variable chains), and the resulting products are transformed into E. coli (DH5-alpha), with colonies selected on LB agar plates containing Hygromycin. At least 16 colonies are selected, and colony PCR is performed to identify positive hits, followed by plasmid purification and Sanger sequencing to confirm the correct clone. The pCP-CMV-GCaMP6f plasmid was purchased from Addgene (#40755). The plasmid was transfected into WT and TMEM16F KO cells using a Lipofectamine LTX kit. G418 was used to select for successfully transfected clones (600 μg/mL).

### Protein expression in Expi293 system

Expi293 cells were cultured in suspension at 37 °C with 8% CO_2_ on an orbital shaker, maintained at a density of 1–2 × 10⁶ cells/mL. Plasmids for the antibody constructs were purified using the Qiagen Endotoxin-free Maxiprep kit and assessed for quality and quantity by Nanodrop. On the day of transfection, the cells were counted by trypan blue exclusion and diluted to a density of ~1.5 × 10⁶ cells/mL with >95% viability using Expi293 expression media. 100 μg of the plasmid DNA and 300 μL of the Expifectamine reagent was diluted in 5 mL of Opti-MEM media in separate tubes. The dilutions were incubated for 5 min at room temperature. The transfection complex was formed by mixing plasmid DNA with Expifectamine reagent in Opti-MEM media and incubating for 15 minutes at room temperature. The transfection complex was added to a 100 mL culture of cells by pipetting slowly and swirling the flask. The cells were incubated for 18 hours, after which transfection enhancers were added. The cell culture was incubated for an additional 72 hours to allow antibody secretion into the supernatant, which was then collected. The cells were collected by centrifugation at 2000 rpm for 10 minutes, and the supernatant was passed through a 0.2 micron filter to remove debris. The protein-containing supernatant was then incubated with 1 mL of Protein-A agarose beads, respectively. The supernatant-affinity bead mixture was incubated for 48 hours at 4 °C.

### Protein purification

After incubating the protein supernatant with beads for 48 hours, the mixture was centrifuged to collect the beads. The supernatant was then passed through a gravity-flow protein purification column containing the beads, with the flow-through collected. For antibody purification, the column was washed with PBS, and elution buffer (0.1 Glycine pH 2.5) was added to elute the antibody in 500 µL fractions, which were neutralized with 0.1M Tris-HCl buffer pH 8.5 and kept on ice to prevent precipitation. The fractions were pooled and concentrated using centrifugal concentrators, then dialyzed overnight in PBS. The purified proteins were aliquoted and kept at −80 °C for long-term storage.

### Antibody and protein labeling

Antibodies were fluorescently labeled using the Dylight 680 or the Dylight 800 NHS ester labeling kit. The Dylight 680 Maleimide reactive kit was used to label the protein containing the Gla domain. To prepare the protein for labeling, a 0.05 M sodium borate buffer at pH 8.5 was used. The protein was dissolved in phosphate-buffered saline (PBS) to avoid precipitation, as buffers containing primary amines like Tris or glycine could interfere with the labeling reaction. For each labeling reaction, 100–500 µL of purified protein at 1–2.5 mg/mL was used, and if necessary, a buffer exchange into the labeling buffer was performed via centrifugal concentrators. For the labeling reaction, the protein solution was transferred to the DyLight 680 NHS-Ester dye, mixed well, and incubated at room temperature for 1 hour. After incubation, excess dye was removed using Thermo Scientific Dye Removal Columns. It was ensured that unreacted dye was completely removed to achieve optimal results. The labeled protein was stored at 4 °C, protected from light, for up to one month, or in single-use aliquots at −20 °C for long-term storage. To calculate the degree of labeling, A280 and the absorbance at A680 were measured using Nanodrop.

### Zr-89 labeling and PET/CT imaging

Conjugation of 11.31 and Isotype antibodies with ^89^Zr was done in accordance with Vosjan and colleagues [71]. Briefly, p-isothiocyanatobenzyl-desferrioxamine (Df–Bz–NCS) was conjugated to lysine groups of the mAbs at pH 9, followed by purification using PD-10 columns [71]. [^89^Zr]-oxalic acid solution was mixed with the Df-Bz-NCS-mAb and incubated at room temperature for 1 h. Unlabeled ^89^Zr was removed by purifying the conjugated antibody using a PD-10 column. The labeling efficiency of the reaction was determined by ITLC. To conduct PET/CT imaging, approximately 200 μg of [^89^Zr]-Df-Bz-NCS-mAb in 200 μL solution was injected intraperitoneally into each mouse before PET/CT imaging with an MILabs VECTor^6^ (Utrecht, Netherlands).

### Scramblase knockouts using CRISPR/Cas9 gene editing

EO771 TMEM16F and Xkr8 KO cells were developed by Synthego using the RNP method. Clones were made by sorting single cells into a 96-well plate using FACS Aria II. MC38 TMEM16F KO cells were created by designing sgRNAs using Genscript. sgRNAs and Cas9-EGFP were co-transfected into MC38 cells using the CRISPR MAX transfection kit. 48 hours after transfection, EGFP was seen in transfected cells and was sorted into single cell clones by FACS Aria II. Clones were screened using Western blot, surveyor assay and q-RT-PCR to validate the knock-out.

Ano6-2-TGGAAATAAGAGTGGACGCG

Ano6-3-TGACACTCTCGTTCACCCGG

Ano-5-CCTTCTCGTAGATCGTGTTG

Xkr8-1-CGTTGAACCAGGAGAAATAG

Xkr8-3-CGTACTGGACAACGGCCCAC

Xkr8-7-CGACCACGTCTAAGGCCACA

### Murine cancer models

8–12 weeks old female C57BL/6 mice (000664) or NSG mice (NOD.Cg-*Prkdc*^*scid*^ *Il2rg*^*tm1Wjl*^/SzJ, 005557) were purchased from Jackson Laboratories. The Rag1 KO mice (C.129S7(B6)-*Rag1*^*tm1Mom*^/J, 003145) were a gift from Bergsbaken Lab (CII, Rutgers University). Homozygous pairs were mated to produce mice for the experiments. Mouse experiments were performed in accordance with the guidelines and under the approval of the Institutional Animal Care and Use Committee at Rutgers University, Rutgers New Jersey Medical School (Newark, NJ). The mice were housed in a pathogen-free facility, maintained under a strict 12-hour light cycle, and given a regular chow diet (5053).

EO771 or MC38 cells were counted and resuspended in DMEM serum-free media at 10^6^ cells/100 μL. The mice were anesthetized using ketamine/xylazine, and the left inguinal mammary gland or the flank was depilated using Nair. 50 μL of the cell suspension was mixed with 50 μL of Matrigel (Corning 354230), and 100 μL of the cell-Matrigel suspension was injected into the mammary gland fat pad or the flank. The tumor volume was measured every 3 days using an electronic caliper, and the tumor volumes were calculated using the formula: Length of the tumor × (Width of the tumor)^2^ × 0.5263. The mice were sacrificed when the tumor reached 2000 mm^3^ or lost 25% of body weight, after which the tumors were dissected, and the tumor weights and spleen weights were measured. Relative tumor volumes were plotted by normalizing tumor volumes at each time point to the WT at endpoint. Mice that did not implant tumors were excluded from the analysis. IVIS imaging was conducted using the IVIS Spectrum. Mice bearing EO771 tumors of ~200–500 mm^3^ were injected intraperitoneally with NIR dye-labeled PS targeting antibodies, and fluorescent imaging was done after 24 and 48 hours after injection. In vivo calcium imaging was done by injecting EO771 cells expressing the GCAMP6F plasmid and waiting for the tumors to reach ~200–500 mm^3^. Mice were fed Alfalfa-free chow (LabDiet 5V75) to minimize background GFP signal in the body.

### Flow cytometry

Cells were collected by trypsinization for adherent cells and by centrifugation for suspension cells. Cells were resuspended and counted by trypan blue exclusion. 1 × 10^6^ cells were collected in a V bottom 96-well plate or a 1.5 mL Eppendorf tube and washed with cell staining buffer (PBS + 1% BSA). Fc block was diluted to 1:50 dilution, and 50 μL was added to cells and mixed by pipetting. Cells were incubated at 4 °C for 20 min. Primary antibody cocktail was prepared, and 50 μL was added to each sample. Cells were incubated at 4 °C for 30 min protected from light. After primary antibody staining, cells were washed with 100 μL cell staining buffer, and then 100 μL of diluted secondary antibody was added and pipetted to mix. Cells were incubated at 4 °C for 30 min protected from light, after which the cells were washed with 100 μL cell staining buffer. For annexin V staining, the BD Apoptosis detection assay kit 556547 was used, and the given protocol was followed. Cytosolic calcium staining was performed in cell culture by treating with 3 μM Fluo-4-AM for 30 min at 37 °C in cell culture and cells were collected and processed for Annexin V staining as mentioned. For fixation, cells were incubated with 4% paraformaldehyde diluted in cell staining buffer for 15 min at room temperature, protected from light. Wherever applicable, cells were permeated using BD Fix perm buffer and stained using active caspase 3 antibody for 30 minutes. Finally, cells were washed with cell staining buffer and resuspended in 300 μL of cell staining buffer and transferred to BD flow tubes. Samples were acquired using BD Fortessa or BD LSR II. Data analysis was done with Flow Jo software.

### Western blotting

Standard SDS-PAGE and wet transfer methods were used for Western blot. Briefly, protein-containing supernatants or cell lysates were diluted in 6X sample loading buffer and subjected to SDS-PAGE (either 8, 10 or 12% resolving gel and a 4% stacking gel) electrophoresis to separate the proteins and further transferred onto a PVDF membrane. The blots were incubated in blocking buffer 5% non-fat dry milk for 1 hour at room temperature, after which they were incubated with the primary antibody β-Actin (CST), p-Akt (CST), TMEM16F(Courtesy Lily Jan, UCSF), Xkr8 (Abclonal) at 1:1000 dilution at 4 °C overnight. The blots were washed thrice with TBST for 15 minutes each, after which they were incubated with an appropriate secondary-HRP antibody (1:4000 or 1:10,000). The blots were washed thrice in TBST for 15 min each, after which they were developed using ECL substrate and imaged using Biorad GelDoc.

### Incucyte assays—proliferation and tumor sphere formation

10,000 cells were plated in a 96-well plate in triplicates. Brightfield images were collected by Incucyte every 3 hours for a total of 72 hours, until cells reached confluency. For the tumor sphere assay, 100 μL of Matrigel (Corning) was coated on the well and cured at 37 °C for 30 min. 1000 cells were embedded into the Matrigel and allowed to form tumor-spheres for ~100 h. Image analysis was done to quantify percentage confluence, counts and size of tumor spheres and plotted as a function of time.

### Efferocytosis assay

Differentiated BMDMs were seeded in a 12-well plate and cultured in IMDM medium containing 0.5% HI FBS. Apoptotic cells were generated by treating Jurkat cells with 1 µM Staurosporine (FUJIFILM) for 3 hours at 37 °C in RPMI medium without serum. After treatment, apoptotic cells were washed and labeled with 100 ng/mL pHrodo Red (ThermoFisher) for 30 min, followed by two washes with PBS containing 1% BSA and 1 mM EDTA, and one wash with IMDM. The labeled apoptotic cells were then resuspended in IMDM supplemented with 10% FBS and added to the plated macrophages at a 3:1 ratio. The co-culture was incubated for 45 minutes at 37 °C. After incubation, macrophages were washed twice with PBS and detached by gentle scraping. Efferocytosis was assessed by flow cytometry, measuring the percentage of pHrodo^+^ cells within the CD11b^+^ F4/80^+^ macrophage population.

### Nanostring RNA profiling

EO771 tumors were excised on day 15 after implantation and flash-frozen in liquid nitrogen. The tumors were digested in Trizol using a rotor-stator homogenizer or the Precellys tissue homogenizer. RNA was extracted using chloroform extraction. The upper aqueous phase was carefully aspirated, and the RNA was precipitated using isopropanol and incubating the mixture at −80 °C for 2 h. The RNA pellet was collected by centrifugation and was washed twice with 70% ethanol. The RNA pellet was resuspended in RNAse-free H_2_O and quality and quantity determined by measuring the OD at A260, A230 and A280 using Nanodrop. The Nanostring gene expression analysis was carried out on the nCounter Max using the Pan-Cancer IO 360 panel by the Rutgers Immune Monitoring and Flow Cytometry Core. The data analysis was done using the Nanostring Rosalind and nSolver platforms.

### Statistical analysis

In vitro experiments were repeated three times. All differences between groups in all in vivo experiments were examined for statistical significance using a two-tailed Student’s *t*-test and two-way ANOVA or Mixed effects model to compare multiple groups. GraphPad Prism software was used to perform statistical analysis, and *p* < 0.05 was considered significant. Power analysis was done to determine the sample size for the mouse experiments.

### Ethics approval and consent to participate

Mouse experiments were performed in accordance with the guidelines and under the approval from the Institutional Animal Care and Use Committee at Rutgers University, Rutgers New Jersey Medical School, Newark, NJ (201702590). Human participants were not used in this study.

## Supplementary information

Supplementary Legends

Supplementary Figures

Original data

## Data Availability

The datasets generated during and/or analyzed during the current study are available from the corresponding author on reasonable request.
