# Supplementary material for: Phospholipid scramblases TMEM16F and Xkr8 mediate distinct features of phosphatidylserine (PS) externalization and immune suppression to promote tumor growth
Source: Cell Death Discov. 2025 Nov 6;11:506. doi: 10.1038/s41420-025-02789-y (PMC12592367; doi:10.1038/s41420-025-02789-y)
Supplement: Supplementary file 1 — Supplementary Legends [file 41420_2025_2789_MOESM1_ESM.docx]

**Supplemental Figures Legends**

**Supplemental Figure 1:**

(S1.A) Schematic showing the antibodies and recombinant proteins used in this study. PS-targeting antibodies 1N11, 11.31, and Bavituximab, as well as a truncated PS-binding modality (GLA+EGF) domain, were expressed and purified for PS binding. (S1.B) Schematic illustrating the experimental setup for tracking PS externalization in EO771 orthotopically grafted tumor-bearing mice using PS-targeting antibodies labeled with near-infrared (NIR) dye or ^89^Zr. (S1.C) IVIS imaging at 24 hours post-injection showing tumor localization of PS-targeting antibodies (11.31, 1N11, Bavituximab and Gla-EGF-Fc) and (S1.D) IVIS imaging at 48 hours post-injection showing continued localization of PS-targeting antibodies in the tumor, while isotype control showed minimal signal. (S1.E) Tissue distribution analysis of Bavituximab at 72 hours after injection, with highest localization observed in tumors and liver, followed by kidney, and minimal in lungs, spleen, and heart. (S1.F) Quantification of Bavituximab localization in tumors compared to other tissues, indicating significant tumor-specific PS exposure (Ordinary One way ANOVA, n=3, * =p<0.05, ** =p < 0.01, *** = p <0.001, **** = p < 0.0001). (S1.G) PET/CT imaging of ^89^Zr-labeled 11.31 antibody at 24 hours post-injection in EO771 tumor-bearing mice, with an isotype control showing lower localization. (S1.H) Quantification of 11.31 localization showing a sustained increase in tumor localization for up to 72 hours compared to isotype control, with liver accumulation observed for both 11.31 and isotype.

**Supplemental Figure 2:**

(S2.A) Kaplan-Meier survival analysis of breast cancer patient data, showing a negative correlation between Xkr8 gene expression and patient survival, with higher Xkr8 levels associated with poorer survival. (S2..B) Kaplan-Meier survival analysis of breast cancer patients, comparing low and high TMEM16F gene expression. Patients with low TMEM16F expression exhibited better survival compared to those with high TMEM16F expression.

**Supplemental Figure 3:**

(S3.A.) Surveyor assay showing a successful KO of Xkr8 gene in EO771 cells. (S3.B.) p-Akt signaling upon treatment of scramblase KO cells with Gas6 shows no significant difference in their intrinsic Axl-Gas6 signaling capabilities. (S3.C.) Quantification of metastatic nodules in the lung from NSG mice injected with scramblase KO tumors. (S3.D.) Incucyte imaging quantifying cell death upon different treatments with the Cytotox dye shows that glucose deprivation and MG132 are not toxic to cells at earlier time points. (S3.E.) Quantification of intracellular calcium and annexin V staining on EO771 cells treated with calcium ionophore shows an early spike with calcium which subsides with time. (S3..F.) Hypoxic conditions for 24 hours with 2% O_2_ did not induce live cell PS externalization in EO771 cells. (S3.G) Schematic of the calcium reporter cell line generated by transfecting EO771 cells with a calmodulin-GFP expressing plasmid to monitor intracellular calcium levels. These cells were implanted into C57BL6/WT mice and IVIS imaging for GFP expression shows both WT and TMEM16F KO tumors harboring high intracellular calcium (circled). (S3.H) Representative flow plots show that Xkr8 KO tumors externalize PS, in response to calcium ionophore treatment as shown by Annexin V staining.
