# Supplementary material for: Phospholipid scramblases TMEM16F and Xkr8 mediate distinct features of phosphatidylserine (PS) externalization and immune suppression to promote tumor growth
Source: Cell Death Discov. 2025 Nov 6;11:506. doi: 10.1038/s41420-025-02789-y (PMC12592367; doi:10.1038/s41420-025-02789-y)

Supplementary Figure 1. *In vivo* localization and biodistribution of PS-targeting antibodies in EO771 tumor-bearing mice.

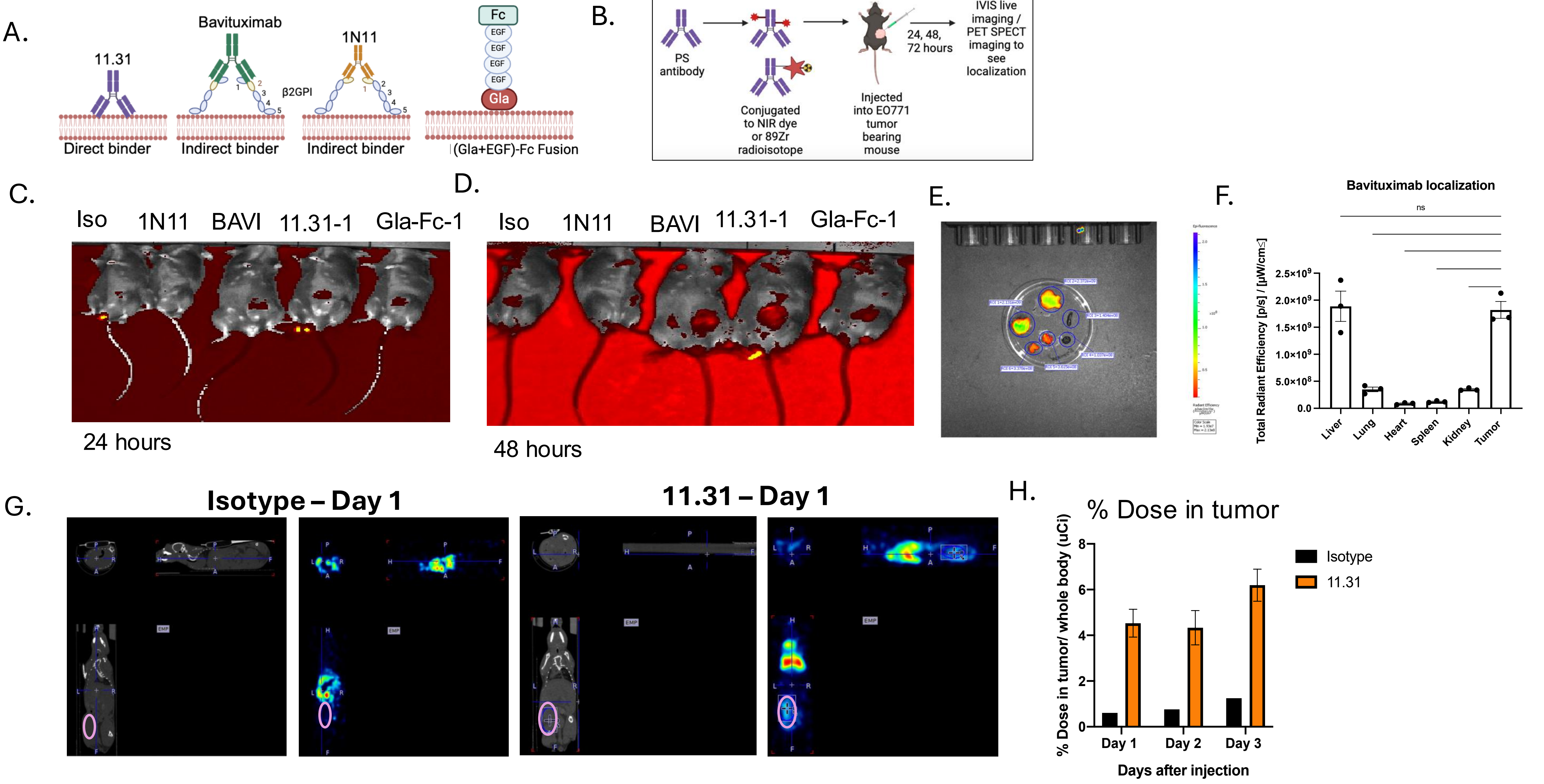

Supplementary Figure 2

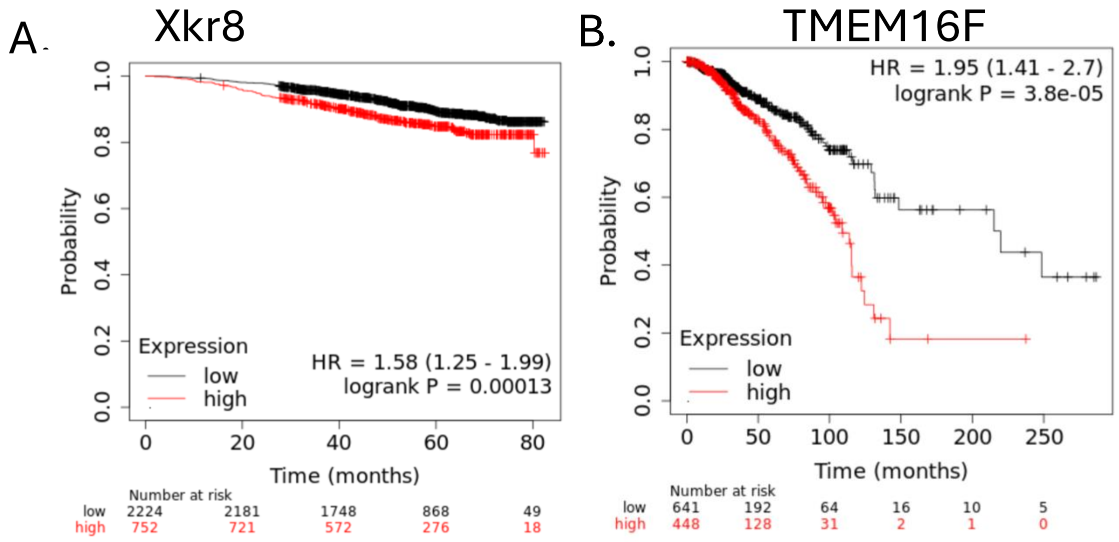

Supplementary Figure 3.

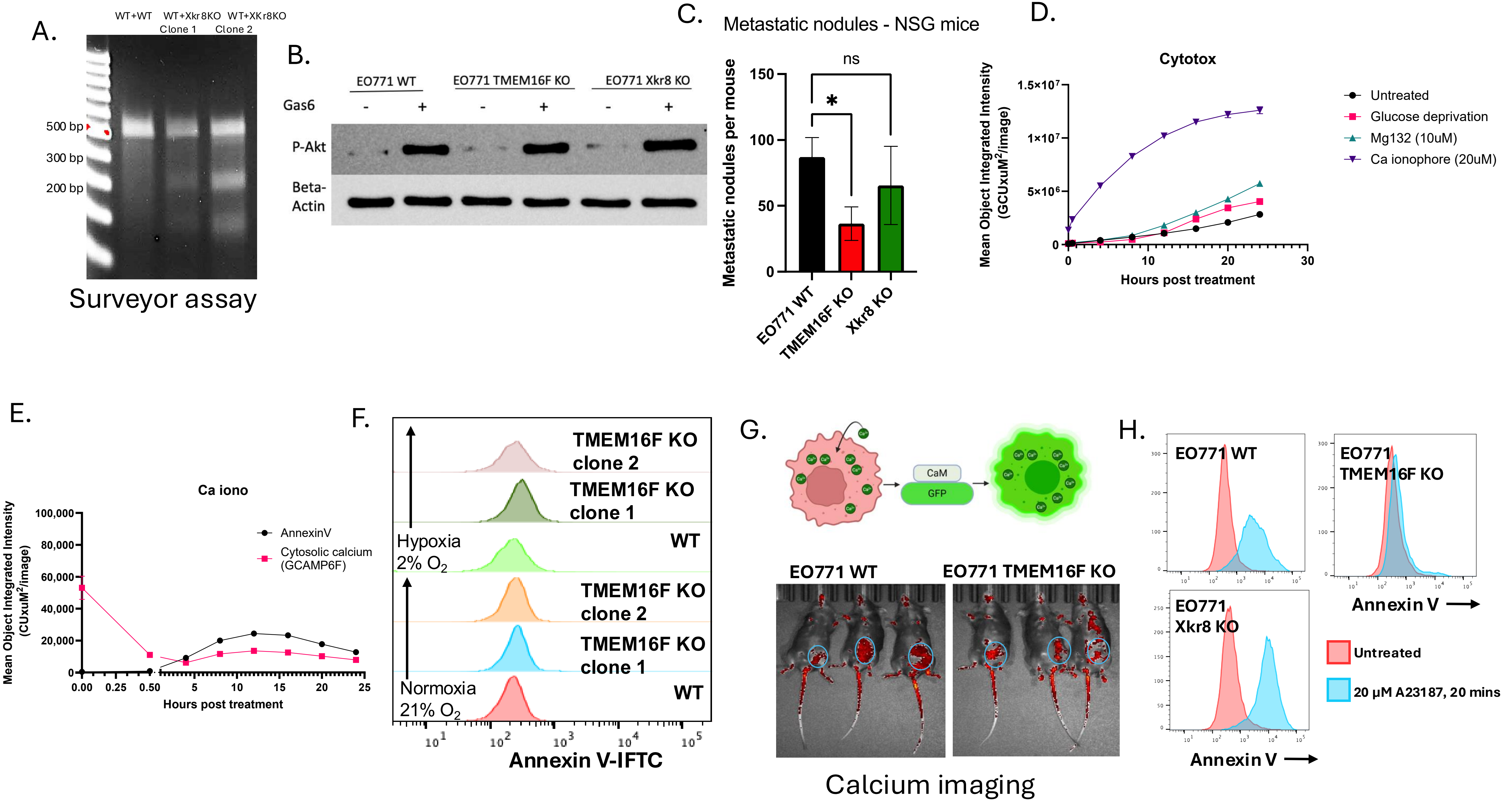

Supplement: Supplementary file 2 — Supplementary Figures [file 41420_2025_2789_MOESM2_ESM.pdf]
