# Supplementary material for: Phospholipid scramblases TMEM16F and Xkr8 mediate distinct features of phosphatidylserine (PS) externalization and immune suppression to promote tumor growth
Source: Cell Death Discov. 2025 Nov 6;11:506. doi: 10.1038/s41420-025-02789-y (PMC12592367; doi:10.1038/s41420-025-02789-y)
Supplement: Supplementary file 3 — Original data [file 41420_2025_2789_MOESM3_ESM.pptx]

## Slide 1
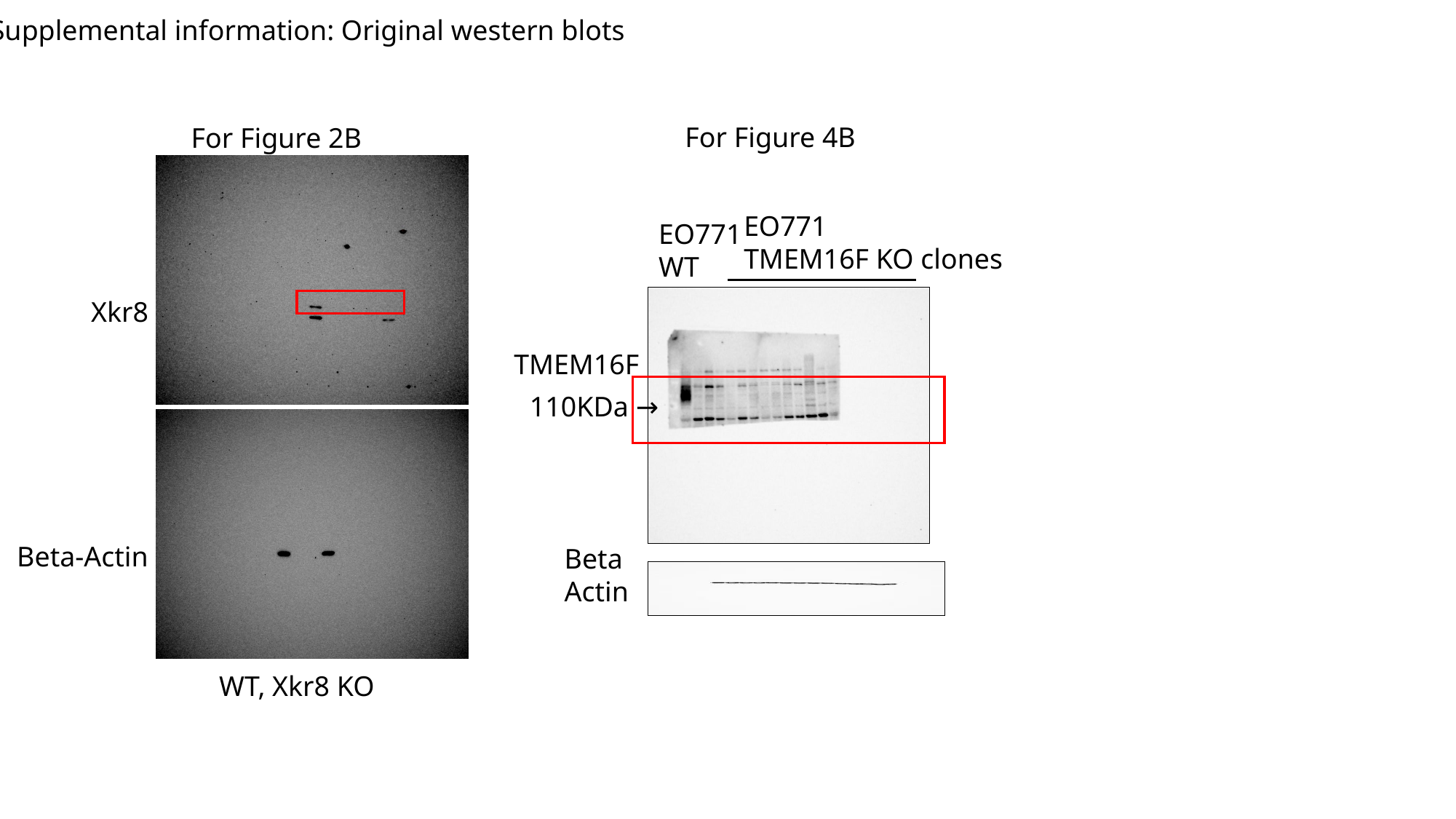

Supplemental information: Original western blots
For Figure 4B
For Figure 2B
EO771
TMEM16F KO clones
EO771
WT
TMEM16F
110KDa →
Beta Actin
Xkr8
Beta-Actin
WT, Xkr8 KO

## Slide 2
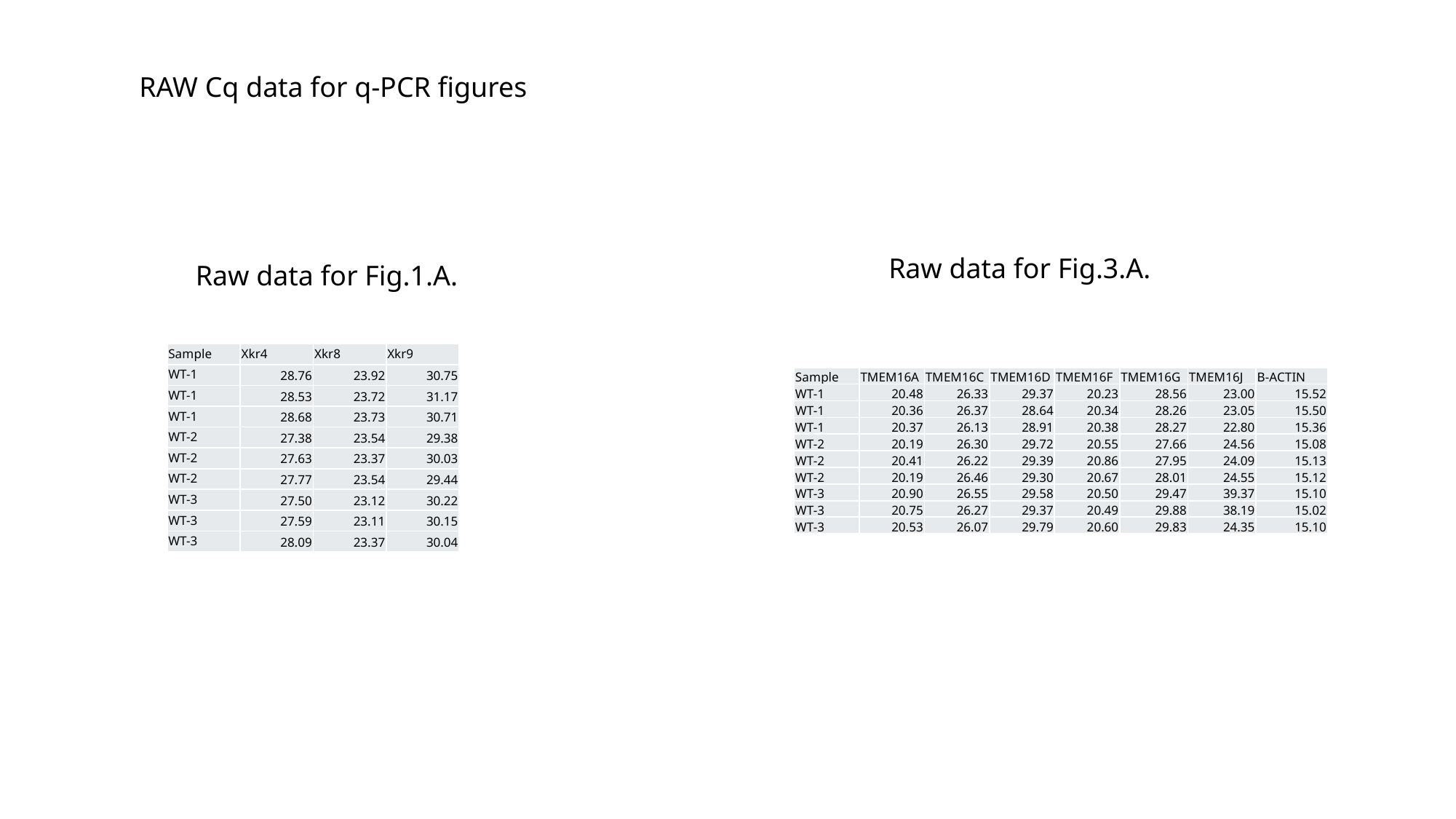

RAW Cq data for q-PCR figures
Raw data for Fig.3.A.
Raw data for Fig.1.A.
| Sample | Xkr4 | Xkr8 | Xkr9 |
| --- | --- | --- | --- |
| WT-1 | 28.76 | 23.92 | 30.75 |
| WT-1 | 28.53 | 23.72 | 31.17 |
| WT-1 | 28.68 | 23.73 | 30.71 |
| WT-2 | 27.38 | 23.54 | 29.38 |
| WT-2 | 27.63 | 23.37 | 30.03 |
| WT-2 | 27.77 | 23.54 | 29.44 |
| WT-3 | 27.50 | 23.12 | 30.22 |
| WT-3 | 27.59 | 23.11 | 30.15 |
| WT-3 | 28.09 | 23.37 | 30.04 |
| Sample | TMEM16A | TMEM16C | TMEM16D | TMEM16F | TMEM16G | TMEM16J | B-ACTIN |
| --- | --- | --- | --- | --- | --- | --- | --- |
| WT-1 | 20.48 | 26.33 | 29.37 | 20.23 | 28.56 | 23.00 | 15.52 |
| WT-1 | 20.36 | 26.37 | 28.64 | 20.34 | 28.26 | 23.05 | 15.50 |
| WT-1 | 20.37 | 26.13 | 28.91 | 20.38 | 28.27 | 22.80 | 15.36 |
| WT-2 | 20.19 | 26.30 | 29.72 | 20.55 | 27.66 | 24.56 | 15.08 |
| WT-2 | 20.41 | 26.22 | 29.39 | 20.86 | 27.95 | 24.09 | 15.13 |
| WT-2 | 20.19 | 26.46 | 29.30 | 20.67 | 28.01 | 24.55 | 15.12 |
| WT-3 | 20.90 | 26.55 | 29.58 | 20.50 | 29.47 | 39.37 | 15.10 |
| WT-3 | 20.75 | 26.27 | 29.37 | 20.49 | 29.88 | 38.19 | 15.02 |
| WT-3 | 20.53 | 26.07 | 29.79 | 20.60 | 29.83 | 24.35 | 15.10 |
